# Self-esteem among People Living with Physical Disability Visting Rehabilitation Centers of Kathmandu, Nepal: An Observational Study

**DOI:** 10.31729/jnma.8801

**Published:** 2024-11-30

**Authors:** Monsoon Jyoti Gautam, Shishir Paudel, Anisha Chalise, Santosh Khadka

**Affiliations:** 1 Department of Public Health, CiST college, Pokhara University, New Baneshwor, Kathmandu, Nepal; 2 Center for Research on Environment, Health and Population Activities (CREHPA), Kusunti, Lalitpur, Nepal

**Keywords:** *disabilities*, *disability studies*, *self esteem*, *Nepal*

## Abstract

**Introduction:**

Self-esteem is a comprehensive personal evaluation of an individual's worth that involves a person's perception of self, it functions as a defense mechanism that individuals possess to safeguard themselves from psychological harm. This study aims to assess the prevalence of selfesteem and its associated factors among people living with physical disability.

**Methods:**

A cross-sectional study was conducted among people living with a physical disability residing inside Kathmandu Valley from August to November 2022 after acquiring ethical approval from institutional review committee of CiST College (Reference number: IRC/161/078/079). A total of 215 samples were taken for the study. A face-to-face interview technique was applied for data collection consisting of the Rosenberg self-esteem scale. Bivariate analysis applying a Chi-square test and multivariable logistic regression was carried out to identify the factors associated with selfesteem at 95% Confidence Interval and 5% level of significance (p-value <0.05).

**Results:**

It was observed that 71 (33.02%) participants had lower levels of self-esteem. Education, family type, the severity of disability, and family affection were the factors associated with self-esteem in bivariate analysis. In multivariable analysis, informal education (aOR: 3.932; 95% Confidence Interval: 1.129-13.696), poor family relationships were (aOR: 2.237; 95% Confidence Interval: 1.029-5.507), perceived severity of disability (aOR: 3.129; 95% 95% Confidence Interval: 1.341-7.300) to be associated with lower self-esteem.

**Conclusions:**

The findings reveal that a significant portion of participants, approximately one-third, experience lower levels of self-esteem. Furthermore, factors such as education, family type, severity of disability, and family affection emerged as significant influencers of self-esteem.

## INTRODUCTION

Physical disability refers to a condition where certain bodily organs or processes exhibit noticeable defects, limitations, or incapacities, leading to hindrances in the execution of ordinary physical movements which hinders various facets of normal life functioning.^1^ Selfesteem is a comprehensive personal evaluation of an individual's worth that involves a person's perception of oneself, it functions as a defense mechanism that individuals possess to safeguard themselves from psychological harm.^2,3^ People living with physical disability feel themselves belonging to marginalized minority groups, often internalizing negative attitudes towards society.^4^ All of this could lead to lower self esteem which could further manifest as psychological distress such as depressive symptoms, anxiousness, or chronic stress in the long run.

In an attempt to better understand the interplay between self-esteem and physical disability, this study aims to assess the prevalence of self-esteem and its associated factors among people living with physical disability in the Kathmandu Valley of Nepal.

## METHODS

This cross-sectional study was conducted among people living with physical disability (PWD) residing inside Kathmandu Valley during the months of August to November 2022. The National Population and Housing Census of 2021 reported almost 2.20% of the Nepali population live with some form of disability of which physical disability accounts for 36.75%.^5^ Kathmandu Valley covers three districts of Nepal, namely Kathmandu, Bhaktapur, and Lalitpur where the proportion of people living with disability lies at 1.50%, 1.40%, and 1.50% respectively.^5^ Kathmandu being the capital of the country and a place with a larger population density and higher proportion of people living with disabilities, it was selected purposively as the study area. The national census of 2021 illustrates a total of 16,042 people with a physical disability residing inside Kathmandu Valley.^5^ The study has considered four rehabilitation centres purposively. There were a population of 485 people living with physical disabilities visiting those four rehabilitation centers. The sample size was calculated using formula:


$$\begin{array}{ll} \text{n} & ={\text{Z}}^{2}\times \frac{\text{p}\times \text{q}}{{\text{e}}^{\text{2}}}\\ & =1.96^{2}\times \frac{\text{0.50}\times \text{0.50}}{0.05^{2}}\\ & =384 \end{array}$$

Where,

Z = Confidence Interval at 95%
p = 50% for maximum sample size calculation
q = 1-p
e = margin of error, 5%

In the absence of prior self-esteem assessments for individuals with physical disabilities, the past prevalence of lower self-esteem was indeterminate so a conservative estimate of 50% prevalence was employed for sample estimation. This gave an estimated sample size of 384, but since the calculated sample population was more than 5% of the total population, finite population correction was applied and the final sample size was established at 215, factoring in a 5% margin of error, and a confidence level of 95%. The required number of samples was drawn using lottery methods from the selected four rehabilitation centers providing rehabilitation and vocational training services to the people living with physical disability inside Kathmandu Valley. To ensure the sample adequacy, the calculated sample of 215 was cross-verified for both bivariate and multivariate analyses using G*Power 3.1.9.^6^ The G*Power software supports sample size and power calculation for various statistical methods (F, t, X2, z, and exact tests).^7^ For the bivariate analysis, a sample estimation for chi-square test was performed with an effect size of 0.30, an alpha of 0.05, a power of 0.90, and a maximum degree of freedom (DF) of 4. The recommended sample size for this analysis was 172, confirming that our sample of 215 was adequate. For the multivariate logistic regression, an effect size (f^2^) of 0.15, an alpha of 0.05, a power of 0.95, and 6 predictors were considered. The G*Power analysis recommended a minimum sample size of 146, further supporting the adequacy of our sample for this model. Furthermore, recommendations from the literature suggest a minimum sample size based on the number of predictors, with proposed guidelines including N>50+p, or N>50+8*p.^8^ Applying these recommendations to our study, which has 6 predictors, the suggested minimum sample size would range from 56 to 98. Given our sample size of 215 exceeds these thresholds, it is considered adequate for the multivariate regression analysis.

Face-to-face interview technique was applied for data collection. The selected participants were approached and provided with study details. The interview was then conducted after obtaining written informed consent from the participants. All the required information from the participants was obtained in a single interview session using a structured interview schedule consisting of the Rosenberg self-esteem scale^9^ to assess self-esteem levels among people living with a disability. Additionally, the interview encompassed other sections designed to comprehensively assess the socio-demographic profiles of the participants.

The Rosenberg self-esteem scale is a 10-item scale where all items are answered using a four-point Likert scale ranging from strong agreement to strong disagreement assessing the self-worth by measuring both positive and negative feelings about the self.^9^ The scale score range from 0-30 where, scores <15 suggest low self-esteem, scores 15-25 suggest normal self-esteem, and scores>25 suggest high self-esteem.^9^ The past study from Nepal noted the good internal consistency of the Rosenberg self-esteem scale translated in the Nepali version where the Cronbach's alpha coefficient was noted to be 0.87.^2^ So, the same pre-tested translated version of the Rosenberg Nepali version was used in this study. To enhance the validity and reliability of the tool, the overall data collection tool was pretested among 20 (10% of the total sample) of the people living with disability, who were not included in the initial sampling frame.

The collected data were carefully reviewed for accuracy and completeness and coded on the same day of the interview. Data were entered in EpiData version 3.1 and 10% of the randomly selected data were manually rechecked for accuracy. The entered data was exported to IBM SPSS Statistics for Windows, version 20 (IBM Corp., Armonk, N.Y., USA). The data were summarised in terms of frequencies and proportions. Bivariate analysis was carried out by applying x^2^ tests to identify the factors associated with self-esteem at 95% Confidence Interval (CI) and 5% level of significance, that is, p-value of <0.05. The variables found to be significant in bivariate analysis were considered for multivariable analysis and included in the final model for multiple logistic regression [for adjusted odds ratio (aOR)] to determine the adjusted effect of each factor on the dependent variable. The multi-colinearity among independent variables was tested using the variance inflation factor (VIF). Hosmer and Lemeshow goodness of fit test was used to assess the goodness of fit of the model.

The ethical approval for this study was obtained from the Institutional Review Committee of CiST College, Pokhara University (Registration no: IRC/161/078/079). The data was collected only after obtaining necessary permissions from the selected rehabilitation institutes and written informed consent from the participants.

## RESULTS

Low self-esteem was seen in 71 (33.02%; 95% CI: 27.2% - 39.8%) and normal self esteem was seen in 144 (66.98%; 95% CI: 60.2%-72.8%) of people living with a disability visiting four rehabilitation centers of Kathmandu Valley. None of the participant reported having higher self esteem.

Out of 215 participants, the average age of the participants was 31.59±8.91 years while the age ranged between 18 to 65 years and 118 (54.89%) were male (Table 1).

**Table 1:** Socio-demographic and disability-related characteristics of participants (n=215).

Variables	n (%)
**Age (Years)**	
20-30	96 (44.65)
30-40	81 (37.67)
40-50	25 (11.62.)
50-65	13 (6.04)
**Sex**	
Male	118 (54.89)
Female	97 (45.11)
**Ethnicity**	
Advantaged ethnic group	98 (45.58)
Relatively Advantaged group	99 (46.04)
Disadvantaged ethnic group	18 (8.38)
**Religion**	
Hindu	152 (70.70)
Buddhist	42 (19.53)
Religious minor	21 (9.80)
**Education**	
Informal	19 (8.83)
Primary	25 (11.62)
Secondary	131 (60.93)
Bachelor and above	40 (18.60)
**Occupation**	
Public service	30 (13.96)
Recreational job	122 (56.74)
Private service	63 (29.30)
**Family type**	
Nuclear	52 (24.18)
Joint/ Extended	163 (75.81)
**Comparison of life**	
Yes	80 (37.20)
No	135 (62.80)
**Severity of disability**	
Mild	56 (26.04)
Moderate	89 (41.40)
Severe	70 (32.56)
**Disability experience**	
Difficult at beginning	114 (53.02)
Hard to deal	101 (47.08)
**Family affection**	
Good	156 (72.55)
Satisfactory	31 (14.40)
Not good	28 (13.02)
**Cause of disability**	
Accidental	130 (60.46)
By birth	19 (8.83)
Due to health condition	66 (30.70)

In the bivariate analysis participants' education status, family type, the severity of disability, and family affection were the factors found to be associated with self-esteem level at a 5% level of significance (Table 2).

**Table 2:** Factor associated with self-esteem in bivariate analysis (n=215).

Variables	Self-esteem		X^2^	p-value
	Low self-esteem	Normal self-esteem		
**Age (Years)**				
20-30	31 (32.30)	65 (67.70)		
30-40	25 (30.90)	56 (69.10)	5.258	0.154
40-50	7 (28.00)	18 (72.00)		
50-65	8 (61.50)	5 (38.50)		
**Sex**				
Male	40 (33.90)	78 (66.10)	0.91	0.763
Female	31 (31.95)	66 (68.04)		
**Ethnicity**				
Advantaged ethnic group	30 (30.60)	68 (69.40)		
Relatively Advantaged group	34 (34.30)	65 (65.70)	0.616	0.735
Disadvantaged ethnic group	7 (38.90)	11 (61.10)		
**Religion**				
Hindu	46 (30.30)	106 (69.70)		
Buddhist	19 (45.20)	23 (54.80)	3.545	0.170
Religious minor	6 (28.60)	15 (72.40)		
**Education**				
Informal	10 (52.60)	9 (47.40)	8.208	0.042^*^
Primary	12 (48.00)	13 (52.00)		
Secondary	40 (30.50)	91 (69.50)		
Bachelor and above	9 (22.50)	31 (77.50)		
**Occupation**				
Public service	14 (46.70)	16 (53.30)		
Recreational job	35 (28.70)	87 (71.30)	3.664	0.160
Private service	22 (34.90)	41 (65.10)		
**Family type**				
Nuclear	24 (46.20)	28 (53.80)	5.347	0.021^*^
Joint/Extended	47 (28.80)	116 (71.20)		
**Comparison of life**				
Yes	22 (27.50)	58 (72.50)	1.757	0.185
No	49(36.30)	86 (63.70)		
Severity of disability				
Mild	11 (19.60)	45 (80.40)	7.612	0.022^*^
Moderate	30 (33.70)	59 (66.30)		
Severe	30 (42.90)	40 (57.10)		
**Disability experience**				
Difficult at beginning	36 (31.60)	78 (68.40)	0.229	0.632
Hard to deal	35 (34.70)	66 (65.30)		
**Family affection**				
Good	45 (28.80)	111 (71.20)	8.604	0.014^*^
Satisfactory	10 (32.30)	21 (67.70)		
Not good	16 (57.10)	12 (42.90)		
**Cause of disability**				
Accidental	40 (30.80)	90 (69.20)	1.020	0.600
By birth	6 (31.60)	13 (68.40)		
Due to health condition	25 (37.90)	41 (62.10)		

* Statistical significance at p<0.05

**Table 3:** Multivariate logistic regression analysis of the factors associated with lower self-esteem (n=215).

Variables	Binary logistic regression			Multivariabl Logistic regression		
	uOR	95% CI	p-value	aOR	95% CI	p-value
Education						
Informal	3.827	1.191-12.293	0.024^*^	3.932	1.129-13.696	0.032^*^
Primary	3.179	1.080-9.362	0.036^*^	2.807	0.904-8.720	0.074
Secondary	1.514	0.660-3.472	0.327	1.734	0.730-4.116	0.212
Bachelor and above	Ref			Ref		
**Family type**						
Nuclear	2.116	1.113-4.020	0.022^*^	1.735	0.861-3.495	0.123
Joint/Extended	Ref			Ref		
**Family relationship**						
Good	Ref			Ref		
Satisfactory	1.175	0.513-2.691	0.704	1.165	0.484-2.804	0.732
Not good	3.289	1.442-7.503	0.005^*^	2.237	1.029-5.507	0.032^*^
**Severity of disability**						
Mild	Ref			Ref		
Moderate	2.080	0.942-4.594	0.070	2.156	0.938-4.954	0.070
Severe	3.068	1.363-6.908	0.007^*^	3.129	1.341-7.300	0.008^*^

* Statistical significance at p<0.05, aOR=Adjusted Odds Ratio, uOR=Unadjusted Odds Ratio, CI=Confidence Interval

The independent variables which were found to have a statistically significant relation with self-esteem in bivariate analysis were subjected to multivariable analysis. Before adjusting the variables in the model, the variance inflation factor (VIF) test was performed to check the multi-collinearity between the variables. The highest observed VIF was 1.85, which suggested there was no multi-collinearity problem. In multivariable analysis, it was observed that in comparison to participants having higher education, those with informal education had a three-fold (aOR: 3.932; 95% CI 1.129-13.696) increase in odds of experiencing lower self-esteem. Similarly, the people living with disability experiencing poor family relationships were twice (aOR: 2.237; 95% CI 1.029-5.507) more at odds of having low self-esteem as compared to those having a good family relationship. Likewise, it was also observed that the participants who perceive their disability to be severe had a three-fold increase in odds (aOR: 3.129; 95% CI 1.341-7.300) of having lower self-esteem than those who perceive it mildly (Table 3).

## DISCUSSION

Drawing upon Morris Rosenberg's definition, selfesteem refers to an overarching self-assessment of an individual's intrinsic value as a human being i.e. their worth as a human being. ^9^ It was observed that two-thirds of people living with physical disabilities had normal self-esteem, while nearly one-third, 71(33.02%) of the people living with disability were experiencing lower self-esteem. While the primary result indicates a significant proportion of individuals maintaining normal self-esteem, the presence of low self-esteem in about one-third of the study population is noteworthy and may still indicate a vulnerable subgroup that warrants attention. Lower levels of selfesteem have been observed among people living with physical disability throughout the world.^4,10-12^ For instance, a study based Korea Employment Agency for the Disabled reported that of 4,033 participants, only 22% participants reported having high self-esteem.^10^ Similar observation was shared by a study based on people with visual disability at Isfahan University of Medical Sciences where the prevalence of low selfesteem was observed to be at 34.8%.^11^ There have been some conflicting observations throughout the world regarding disability status and self-esteem. In some of the studies, it has been observed that people with physical disabilities have significantly lower self-esteem than those without any disabilities.^4,13-16^ However some studies have concluded no significant difference exists between self-esteem and the presence or absence of disability.^17^

The impediments resulted due to physical disability restrict an individual's ability to perform essential daily activities, giving rise to dependency and a diminished capacity for autonomous functioning.^4^ This could create a sense of stigmatization among the people living with such hardship. When self-esteem is contextualized in regards to disability, the person living with a disability could assign his or her capacity to integrate within a society and function accordingly as a sense of self-worth.^18^ For people living with disabilities, the experience of disability often intersects with societal stigmatization, potentially eroding their self-perception. Thus, self-esteem is an important psychological factor contributing to health and quality of life and a significant etiological agent for mental distress.^19^ Low self-esteem could pose a considerable threat to the person's efficacy, competence, learning, and creativity. This phenomenon is characterized by sentiments of inadequacy, guilt, reticence, social inhibition, hopelessness, withdrawal, reduced competence, and a tendency to downgrade others.^4^ It had been linked as a risk factor for several emotional, psychological, and behavioral problems.^20^ Thus, considering their vulnerability towards negative selfassessment, people living with disabilities should be aware of cultivating healthy self-esteem.^12^

Mostly the variation in these observations has been attributed to social activeness, education, and empowerment status of the people living with physical disability. In this study, it was also observed people living with disability, who didn't have formal education were thrice more likely to experience lower self-esteem in comparison to those with higher education.

It was observed that the severity of disability has a statistically significant relation with self-esteem among PwPD having low self-esteem. This is in line with past studies suggesting that disability severity can cause frustration and distress among individuals leading to lower self-esteem.^21^ Similarly a study based on disabled adults in South Korea suggests that people who accepted their disability were twice more likely to have higher self-esteem than the ones who could not accept their disability status.^12^ In a study examining self-esteem among active and inactive people with physical disability it was observed that active PWPD had higher self-esteem in comparison to inactive PwPD.^22^ All of these findings suggest that education could empower people with physical disabilities. Their acceptance of their limitations and empowerment for active engagement in social and economic sectors could boost their self-worth, leading to better self-esteem.^18,23^ All of these suggest a complex interplay exists between education, acceptance, activity, and self-esteem among people with physical disabilities.

It was observed that good family relationship is crucial for better self-esteem among people with disability as those with poor family support were twice more likely to have lower self-esteem. This is in line with a past study from Nepal where those women who were living with a disability and felt themselves as a burden to the family, rejected and excluded from the family group could experience lower self-esteem.^24^ Similarly, it has also been observed that people who perceived their disability to be less, have positive school, and home environment, less over-protection and greater family support could have higher self-esteem.^16^ Selfesteem has been observed to have positive relations with social support among people with physical disabilities.^25^ These observations suggest that strong family relationships is a key to higher self-esteem and underscore the importance of fostering supportive family environments and inclusive communities to boost the well-being of individuals with disabilities.

Despite being one of the few studies assessing selfesteem among people with physical disabilities in the Nepalese context, this study is not free from its limitations and all the results presented should be interpreted considering these limitations. Due to the unavailability of the complete list of people living with physical disability inside Kathmandu Valley, the study was executed in selected rehabilitation centers. Given its institution-based nature, we failed to cover the PwPD residing in the communities and not listed in the rehabilitation centers which might have introduced some selection bias, and a slight risk of underrepresentation of lower self-esteem among PwPD in reference to community setting. Thus, a larger community-based survey covering these active as well as inactive people living with a disability might provide additional insight into self-esteem and its contributing factors among these vulnerable population subgroups.

## CONCLUSIONS

Nearly one-third of participants in this study with physical disabilities reported lower self-esteem. This study identified education, family support, and perceived severity of disability as major factors associated with low self-esteem, revealing a potential existence of complex interplay between these factors.
